# Recent Modalities in Pain Control and Local Anesthesia in Dentistry: A Narrative Review

**DOI:** 10.7759/cureus.48428

**Published:** 2023-11-07

**Authors:** Abhishek Pahade, Pavan Bajaj, Unnati Shirbhate, Hussain Ali John

**Affiliations:** 1 Dentistry, Sharad Pawar Dental College, Datta Meghe Institute of Higher Education and Research, Wardha, IND; 2 Periodontics, Sharad Pawar Dental College, Datta Meghe Institute of Higher Education and Research, Wardha, IND

**Keywords:** local anesthesia, anesthetic agents, nsaids, lignocaine, management, pain control

## Abstract

Pain in the orofacial region or within the tooth is one of the most common complaints patients report to a dental office. An efficient practitioner must have adequate knowledge and tools to address and remedy the problem. Pain control in dentistry has a rich history and learning about it gives an insight into how the current modalities being used came into existence. As dentistry keeps evolving, newer and more efficient modalities have been developed for pain control. Dental pain is primarily remedied by dental practitioners and clinicians involved in emergency medicine; it may result due to various causes, mainly insulting the tooth or complications involved in and after oral surgery. Several modalities have been developed to reduce and eliminate this, including pharmacological and non-pharmacological treatment modalities. Pharmacological modalities include using drugs. Many medications are used for pain management, such as non-steroidal anti-inflammatory drugs, corticosteroids, and muscle relaxants. Non-pharmacological modalities include behavior control methods based on several theories of pain. These modalities are used mainly for children, but some can also be used for adult patients. Several advances in delivery systems for local anesthesia involve using newer technologies to deliver a sustained dose of anesthetic agent. This review aims to enlist both modalities of pain control management in dental practices along with the newer advancements in this field.

## Introduction and background

According to the International Association for the Study of Pain (IASP), “Pain is an unpleasant sensory and emotional experience associated with actual or potential tissue damage or described in terms of such damage.” This definition is widely accepted worldwide by practitioners, healthcare professionals, and public health activities. It has also been adopted by several governmental and non-governmental organizations, including the World Health Organization (WHO) [1]. Several other definitions also exist; one of the popular ones is by Monheim who defined pain as “an unpleasant emotional experience usually stimulated by a noxious stimulus and transmitted over a specialised neural network to the Central Nervous System (CNS), where it is interpreted as such” [2]. Pain has a wide range of severity, quality, duration, and various pathophysiologic causes and meanings. The current IASP definition recognizes that, while tissue injury is a common cause of pain, it can be present even when tissue damage is not visible [1,2].

As pain is one of the most common etiologic factors for which patients seek dental treatment, a practitioner must have the proper knowledge to deal with the problem adequately. Management of pain in dental practice following the “Three-Dimensional” (3-D) principle has been suggested, which involves diagnosis, dental treatment, and the use of drugs. The first step in managing dental pain is diagnosis. The patient must be examined clinically and through various kinds of investigations available to specify the etiological factors or agents, enabling the practitioner to formulate a proper treatment plan [3]. Multiple clinical tests can be performed with the help of instruments, such as the percussion test. Advanced modalities, such as radiographs, must be employed to screen the patient for the cause of pain. Only when the actual cause of pain is identified can the practitioner use modalities to treat the patient effectively. The second step is dental treatment which results in the resolution of the symptoms experienced by the patient. After this, drug therapy can be initiated, and the choice of drugs usually depends on the symptoms experienced by the patient and the treatment modalities employed to treat the cause [3,4]. If the cause of a sickness or condition cannot be determined, the diagnosis is incomplete. The reason could be simple (such as pulpal diseases and carious teeth) and require standard dental management, or it could be complex (medical reason) and require treatment other than dental care. Identifying the origin of the disease is important as removing the primary etiology is an essential aspect (and in the initial stage) of disease management [3].

## Review

Pathophysiology and etiology of orofacial pain

The trigeminal nerve and the supporting structures supply various structures in the orofacial complex, including the mouth, face, and scalp. This region is very complex and involves multiple systems involving the eyes, ears, cheeks, etc. The trigeminal nerve is the fifth cranial nerve; it is mixed in nature and is responsible for both motor and sensory functions. Due to the parts it supplies, it is also called the dentist’s nerve. The trigeminal nerve is divided into three branches, namely, V1, or ophthalmic division; V2, or the maxillary division; and V3, or the mandibular division, with V3 being the most extensive [5].

Many pathological conditions and diseases can cause orofacial pain, and these may be related to the somatic or neural structures of the body. The conditions that may cause pain in the orofacial region, fascinating to a dental practitioner, are the hard and soft tissue. Dental pain is primarily remedied by dental practitioners and clinicians involved in emergency medicine. It may result due to various causes, mainly insulting the tooth or complications involved in and after oral surgery [6,7]. Odontogenic pain is defined as pain that originates in the mucosa, gingivae, maxilla, mandible, or periodontal membrane or in the teeth or the tissues that support them. On the other hand, inflammation caused by trauma, infections, or tumors can result in non-odontogenic facial pain. Dental caries is the most prevalent cause of toothache, which is initiated by dental pulp inflammation. The second most prevalent infection is periodontal disease, often known as gum disease, which is painless, such as chronic Mycobacterium infections like leprosy. Aggressive periodontal disease is especially likely to be caused by these two bacteria. Multiple deep gum pockets and resistance to conventional gum disease therapies are linked to *Porphyromonas gingivalis* and *Actinomyces actinomycetemcomitans* [7]. Dental diseases associated with orofacial pain are enumerated in Table 1.

**Table 1:** Dental diseases associated with orofacial pain. [6,7].

Involving hard tissue	Involving soft tissue
Dental caries involving enamel/dentin/cementum	Pulpitis
Dentin hypersensitivity	Gingivitis
Dry socket	Periodontitis
Osteomyelitis	Pericoronitis

Pharmacological management

Analgesic medication, administered orally or parenterally, preoperatively or postoperatively, is an integral part of pain management in dentistry [8]. Mainly, two drugs used in dentistry for acute pain management are non-steroidal anti-inflammatory drugs (NSAIDs) and opioids. The use of opioids is usually restricted due to their addictive nature. The three most commonly used drugs in dentistry are aspirin, ibuprofen, and paracetamol. These drugs are also readily available and widely used in medicine, called over-the-counter drugs. Corticosteroids have also been used sporadically for pain management in dentistry [5,8].

Pain that is neural in origin can also be managed effectively using anticonvulsants or antidepressants such as amitriptyline. Tricyclic antidepressants (TCAs) used earlier had a higher potential to cause cardiovascular and CNS toxicity, but they had greater efficacy; for this reason, they are not preferred in patients suffering from heart conditions or liver disease [3]. Drugs such as tramadol are also highly effective but can cause seizures, dizziness, and confusion. Drugs such as gabapentin have also proven to be well tolerated by patients; however, they are associated with mood swings and depression [9]. For managing pain associated with the temporomandibular joint, using the class of drugs known as muscle relaxants has proven to be an excellent choice. Analgesic properties have been demonstrated with this class of drugs. Baclofen and cyclobenzaprine are some examples. Cyclobenzaprine is usually the choice of drug for patients with bruxism [10,11].

Fever and other inflammatory diseases can be treated with NSAIDs. NSAIDs are categorized as either selective cyclooxygenase (COX)-2 NSAIDs or non-selective COX inhibitors. Based on their chemical structures, acetylated salicylates, non-acetylated salicylates, propionic acids, enolic acids, and anthranilic acids can be categorized as non-selective COX inhibitors [6,12,13]. The classification of NSAIDs is presented in Table 2.

**Table 2:** The classification of NSAIDs. [6,12,13]. NSAIDs: non-steroidal anti-inflammatory drugs; COX: cyclooxygenase

Non-selective NSAIDs	COX-2 selective NSAIDs
Diclofenac	Celecoxib
Diflunisal etodolac	Rofecoxin
Indomethacin	Valdecoxbin
Fenoprofen	
Flurbiprofen	
Ketorolac	
Piroxicam	
Naproxen	

Ibuprofen

Pharmacologically, ibuprofen is a 2-propionic acid derivative with good analgesic and anti-inflammatory action. It has been extensively studied and is frequently used in dentistry, with several studies supporting its potent activity. It exists as a racemic mixture. The pharmacokinetics of this drug do not depend on the patient’s age [13]. It is rapidly absorbed from the gastrointestinal (GI) tract and available in immediate and sustained-release forms. It has a short half-life of about 2.1 hours. Ibuprofen is metabolized in the liver via the cytochrome enzyme; however, if the patient is suffering from any liver condition such as liver cirrhosis, it can be affected. COX is required for the production of prostacyclins, prostaglandins, and thromboxanes from arachidonic acid. Ibuprofen inhibits cyclooxygenase, thus preventing further processes [14,15].

Aspirin

Aspirin has been used as an effective pain medication for many years and is used for moderate-to-severe pain. The pain relief achieved by aspirin is very similar to paracetamol. However, it has significant side effects, such as gastric irritation and drowsiness [16,17]. For moderate-to-severe acute pain, aspirin works well as an analgesic. Higher dosages yield better results. Striking similarities were noted in comparing aspirin’s pain alleviation to paracetamol’s, milligram for milligram [16].

Paracetamol

Paracetamol has excellent analgesic action but poor anti-inflammatory activity. Three is the number-needed-to-treat (NNT) and thirty-three is the number-needed-to-harm for 1,000 mg of paracetamol at six hours. The NNT offers a means of measuring the efficacy of a drug or therapy by estimating the number of patients who need to be treated to affect one individual. After oral surgery, paracetamol works well as a postoperative pain reliever, and reports of side effects indicate that the drug is safe. It works best at doses of 1,000 mg and can be taken every six hours [18]. Patients and dentists may be more receptive to it when used alone or in combination with other analgesics, such as NSAIDs, to reduce pain. The use of paracetamol and its efficacy has been well established, but it has also been reported that it can be misused, resulting in overdose and hepatotoxicity [19].

Local anaesthesia

According to Malamed, “local anaesthesia is defined as a transient loss of sensation in a circumscribed area of the body caused by depression of excitation in nerve endings or an inhibition of the conduction process in peripheral nerves” [20]. Several agents have been used over the years to produce local anesthesia. The application of local anesthesia in dentistry is usually topical or through injectional blocks. Two aspects determine how long a local anesthetic lasts, i.e., whether it binds to proteins or gets redistributed.

Topical anesthetics are agents that cause numbness of the mucosa or the overlying skin; they work by blocking the conduction of the superficial nerves on the surface. These are usually used in mild conditions, such as minor aphthous ulcers, to reduce the pain and discomfort of injections, or in invasive procedures. Topic anesthetics are available in various forms, including sprays, ointments, creams, gels, and solutions. They may also be flavored with agents such as mint, cherry, and bubble gum. The concentrations at which topical anesthetic agents are used are usually higher than those used in injectable agents, but the onset of action is poor compared to injectable ones [20,21]. The agent’s placement should be on a clean, dry surface to maximize its effect. The most famous agent is 20% benzocaine. The United States Food and Drug Administration (USFDA) recommends a combination of 18% benzocaine and 15% tetracaine, which has proven to be more efficacious. Custom-made medications can be prepared by combining two ingredients, which is known as compounding. These have more efficacy and have a high concentration of the anesthetic agent and are usually used in pediatric patients, for periodontal procedures, and by orthodontists for placing temporary anchorage devices [21]. EMLA is a eutectic mixture of local anesthesia, which is a typical example; it contains 2.5% lignocaine and 2.5% prilocaine. These two ingredients are mixed to form a eutectic mixture. It is supposed to be used on intact skin and is available in gel form [22].

Injectable Anesthesia Agents

Benzocaine is a poorly soluble ethyl ester of P-aminobenzoic acid and one of the ester local anesthetics used in topical as well as in injection form. Benzocaine is available in five forms, i.e., aerosols, gel, gel patch, ointment, and solution. These forms can be employed in various situations [21]. For example, aerosols might be used for the administration of palatine anesthesia in patients while obtaining impressions; the gel form of benzocaine is used before the application of local anesthetic injections to minimize injection pain and discomfort; and ointment and solution form of benzocaine typically provide comfort in patients with oral aphthous ulcers [23]. Prilocaine is another amide regional anesthetic commonly used in dentistry for anesthetic infiltration. Because it is a secondary amide, it differs from lignocaine and mepivacaine. Because of its lower systemic toxicity and broadly categorized therapeutic range, articaine is used at larger concentrations than other amide local anesthetics [21-23]. Articaine diffuses through bone and soft tissue better than other local anesthetics, a unique characteristic that makes it an appealing local anesthetic. Bupivacaine is frequently used after removing impacted third molars to relieve discomfort for at least 12 to 20 hours. It is also often injected into surgical incision sites to relieve long-term pain. Adrenaline, or epinephrine, is a sympathomimetic amine used in local anesthetics to produce vasoconstrictor effects [21]. Classification of local injectable anesthetic agents based on the chemical structure is presented in Table 3 [21-23].

**Table 3:** Classification of injectable local anesthetic agents based on chemical structure. [21-23].

Amino acids	Amino esters
Lignocaine	Procaine
Bupivacaine	Cocaine
Mepivacaine	Benzocaine
Etidocaine	Tetracaine
Ropivacaine	Chloroprocaine
Levobupivacaine	

Lignocaine

One of the oldest anesthetic agents is lignocaine/lidocaine. It is an amide and has been used for more than 60 years. It is metabolized in the liver and excreted through the kidneys. Lignocaine is one of the most commonly used injectable anesthetic agents used in dental offices; however, it has specific contraindications, such as severe liver disease, allergy to lignocaine, heart block, and patients on antiarrhythmic therapy [21,23,24]. The European Pharmacopoeia recommends a maximum dose of 200 mg of lidocaine without adding epinephrine. In comparison, the USFDA recommends a maximum dose of 300 mg. There might need to be more than this amount of standard lidocaine for regional anesthetic operations in many adults [25]. The maximum recommended dose for adults for lignocaine with epinephrine is 7 mg/kg up to 500 mg. Lignocaine without epinephrine is 4.4-4.5 mg/kg up to 300 mg [21,25].

Non-pharmacological modalities

Non-pharmacological modalities work on the principle of behavior management, which is based on several theories of pain. These help reduce patient anxiety, which is very important as anxiety lowers the pain threshold and makes the patient uncooperative. These modalities are extensively used in pediatric patients; however, some might also be used in adult patients. The tell-show-do technique, music therapy involving white noise to relieve stress, plant extracts, acupuncture, ice therapy, transcutaneous electrical nerve stimulation, and relaxing breathing exercises are some of the modalities used [26,27]. Kojima et al. described ultrasound-guided nerve blocks to curb postoperative pain in patients with maxillofacial surgeries [28].

History

The modern dental syringe was invented by Cook about 150 years ago, even though traditional aspirating needles are still more widespread [29]. The perception of pain is a part of human life; even though humans are not the only beings that can feel pain, their perception is essential. It is an unpleasant and necessary stimulus because if the perception of pain was absent in humans, noxious agents would cause harmful effects on the body. Earlier, it was believed that pain was caused by demons, evil spirits, or magic fluids, but as humans evolved, modern theories were proposed [30]. In the Netherlands, pain control is usually done by surgeons; since 1846, anesthesiologists and dentists have been working hand in hand to provide the best and pain-free treatment to the patients, resulting in less trauma and increased patient satisfaction [31].

Recent advances

Even though the standard aspirating syringe is still the most popular way to administer local anesthetics, more recent technologies have been developed to help the dentist provide more significant pain relief with fewer painful injections and side effects. Vibrotactile devices are the newer local anesthesia delivery systems that use the gate control theory, which is the theory of pain that proposes that pain can be lessened by nerve fiber activation through vibration [29,32]. VibraJects are small attachments that can be fixed in the syringe. It helps deliver a sustained vibration that the patient can feel; it is a battery-operated attachment, and its use has been controversial as studies about its efficacy have been mixed [20,33]. DentalVibe is a system that works on the gate control theory principle. It is a battery-operated hand-held system that produces micro-oscillations stimulating sensory receptors at the injection site, which helps block the painful sensation of injections [29,34]. Accupal is a cordless gadget that preconditions the oral cavity’s mucosa by vibration and pressure. It shuts the pain channels around the injection site, 360 degrees proximal to the point of needle penetration. It is battery-operated and uses a AAA battery [29,35]. Computer-controlled local anesthesia delivery systems are a newer modality incorporating computer technology to control the anesthetic agent’s flow rate through the needle. A controlled flow would ensure only an adequate solution is injected, preventing complications such as overdose and toxicity [36]. Jet Injectors use mechanical energy to push a solution through a small orifice forcefully. This can penetrate soft tissues that use a needle and can help eliminate patient anxiety and fear. Examples are Syrijet and Med-Jet [33,37].

Safety Dental Syringes

There are several hazards a practitioner may face when working in a dental office, with one of the hazards being accidental needle-stick injury. To eliminate this hazard, safety dental syringes have been introduced. These have a sheath that locks over the tip of the needle after administering an anesthetic agent to the patient. Hence, the chance of injury is reduced. Safe dental syringes have been recommended by the Centers for Disease Control and Prevention. Examples are the Ultra Safety Plus XL Syringe by Septodont, UltraSafe syringe by Safety Syringes, HypoSafety Syringe, SafetyWand, and RevVac safety syringe [33,38]. Local anesthesia has come a long way in pain management. However, there is more scope for improvement to make the dental experience painless and enhance patient satisfaction [29].

## Conclusions

Pain is essentially a human experience of perception, although many other species do experience pain. Pain helps humans prevent noxious stimuli from causing damage. In dentistry, pain management includes various procedural concerns such as anesthetic delivery and postprocedural pain management. It also includes pain diagnosis, management techniques for orofacial conditions that cause pain in the head and face, and pain management for specific populations. Pain is common during and after dental procedures, and several modalities have been developed to reduce and eliminate pain. The modalities that exist can be classified as pharmacological and non-pharmacological modalities. Pharmacological modalities include using drugs, even though many medications are used for pain control, such as NSAIDs, corticosteroids, and muscle relaxants. Local anesthesia is a popular modality used in topical or injectable forms. Non-pharmacological modalities include behavior control methods based on several theories of pain. These modalities are used mainly for children, but some can also be used for adult patients. Several advances in delivery systems for local anesthesia involve using newer technologies to deliver a sustained dose of anesthetic agent.
